# Determination of Osmotic Flow in Water Transport in an Illitic Clay

**DOI:** 10.3390/ma18020338

**Published:** 2025-01-13

**Authors:** Marek Mánik, Igor Medveď, Martin Keppert, Zbigniew Suchorab, Anton Trník

**Affiliations:** 1 Department of Physics, Faculty of Natural Sciences and Informatics, Constantine the Philosopher University in Nitra, Tr. A. Hlinku 1, 94901 Nitra, Slovakia; mmanik@ukf.sk; 2 Department of Materials Engineering and Physics, Faculty of Civil Engineering, Slovak University of Technology in Bratislava, Radlinského 11, 81005 Bratislava, Slovakia; igor.medved@stuba.sk; 3 Department of Materials Engineering and Chemistry, Faculty of Civil Engineering, Czech Technical University in Prague, Thákurova 7, 16629 Prague, Czech Republic; martin.kepppert@fsv.cvut.cz; 4 Faculty of Environmental Engineering, Lublin University of Technology, 40B Nadbystrzycka Str., 20-618 Lublin, Poland; z.suchorab@pollub.pl

**Keywords:** osmosis, molar flow, transport coefficients, porous media

## Abstract

Experimental studies have shown that osmosis could be one of the mechanisms of water transport in porous materials that act, to a certain extent, as semipermeable membranes. In this paper, an experimental apparatus and the corresponding model to measure and determine the osmotic efficiency, *σ*, of bulk porous materials are described. Both the apparatus and model to interpret water transport in samples are modifications of those of Sherwood and Craster. In addition to *σ*, the transport parameters of the model include Darcy permeability and water and salt diffusivity. These parameters are used to calculate the ratio of the individual components of the total molar flow. We used the apparatus to measure cylindrical samples made from an illitic clay with a diameter of 45 mm and thickness of 5 mm. The measured transport coefficients were then used to estimate the relative importance of the individual contributions to the total flow of water through the samples. Our results show that the contribution of the osmosis is 82–88%, while the diffusion contributes only 11–13% and the Darcy flow caused by the pressure difference contributes only 1–5%. Even after considering the uncertainties in the measurement of the transport coefficients, which are estimated to be up to 22%, the results show that osmosis makes an important contribution to the total water flow and should not be neglected in general.

## 1. Introduction

When a porous medium separates a solution of two different concentrations, a gradient in concentration is always present. Such a system tends to neutralize this gradient by moving either the solvent or diluted particles through the medium. Hence, a flow through the medium is observed. Such a flow is very often identified with diffusion. However, other mechanisms, including osmosis (which is a passive transport of solvent species through a barrier that restricts the transport of solute species), may also have a noticeable contribution to the overall transport in some cases. Namely, osmosis can occur if the porous medium has selectively permeable properties which may be present for various reasons [1], such as the size of the micropores or the innate electromagnetic properties of the material. Indeed, the micropores in the material can have micropores of a specific size that allows the free flow of small molecules (like water), but bigger particles (like salt ions) cannot fit in, and therefore, they are restricted [2]. Moreover, the intrinsic electromagnetic properties of the material either bind or repel electrically charged ions while allowing a flow of electrically neutral particles [3].

Various porous materials have been observed to be selectively permeable, at least to some extent. For example, for natural clays, this was experimentally confirmed in the laboratory [4,5] as well as in field experiments [6]. Such clays have a layered structure (double- or triple-layered) with charged particles near the edges of the layers [7,8]. These particles are responsible for low electrostatic fields in the interlayer space which can restrict the transport of charged particles (i.e., salt ions), while the electrically neutral water molecules can flow without any hindrances [3,9]. Thus, although the transport of water may occur mostly due to diffusion through the pores in the clays, osmosis through these interlayer spaces could also be relevant. Systematic investigation of osmosis in porous materials and the extent of its relevancy in water transport is still lacking. That is perhaps why most of the recent studies of osmosis through porous media use their own model of the osmotic transport and an experimental setup adapted specifically to their needs. For example, Sherwood and Craster studied an osmotic transport of water and salt ions through a porous membrane made of montmorillonite films that contain glassy beads [10]. They conducted an experiment with KCl solutions of different concentrations and showed that osmotic transport occurred in this material, with the osmotic efficiency (also known as the reflection coefficient) being rather low: *σ* = 1.1 × 10^−3^. Kooi and his coworkers conducted numerical experiments and compared them with measured data obtained by long-term in situ experiments in South Dakota [11,12,13], determining the osmotic efficiency to be *σ* = 0.089. Simulations show that osmotic efficiency varies greatly with the distance from the edge of the sample; at the edges of the sample *σ* ≈ 0.05, but deeper in the sample, it increases dramatically, reaching *σ* ≈ 0.95 near the sample center [14]. Other studies point out changes in the osmotic properties of natural clays based on additives used in the production process. Their results show that the osmotic efficiency of natural clays is rather low, its value being *σ* ≈ 0.01, but it can increase even up to *σ* ≈ 0.97 via the addition of bentonite or smectite in the samples [15]. Osmotic properties are present also in clay minerals that contain salts [16] and bitumen waste [17]. Even though *σ* is usually expected to have values between 0 and 1, representing no osmotic properties and an ideal membrane, respectively, some experiments resulted in anomalous values, both negative and positive [18]. Such behavior was explained by the influence of electro-osmosis induced by the diffusion. Another study of kaolin–bentonite mixtures showed that *σ* varies from 0 to 0.197, its value increasing with the amount of bentonite [19]. This study also suggests the existence of a threshold bentonite content above which osmotic properties cease to improve. Some studies indicate that using the van ’t Hoff equation to calculate the osmotic pressure may give incorrect results, because it considers a membrane to be ideal. Instead, different models were proposed to calculate the osmotic pressure based on the activity of water instead of an average chemical potential of solutions [20,21]. In both cases, it is shown that using activity of water yields higher values of *σ* compared to the van ’t Hoff equation. Even though the studies usually observe the behavior of a membrane exposed to a solution with only one type of ion, in real applications, it is usually exposed to a wide variety of ions. Zhang and co-authors therefore proposed a model to calculate the osmotic efficiency in more variable environments in which a clay membrane is exposed to mixed solutions with multiple types of ions [22,23]. An interest in the evaluation of chemico-osmotic properties appeared in studies of diffusion in geosynthetic clay liners, both unused [24,25] and exhumed from a landfill after 12 years of use [26]. Their containment properties, improved by the presence of chemical osmosis, are suitable for use as a barrier for the separation of municipal waste from the environment. In [25], a testing apparatus for assessing chemico-osmotic and diffusive properties of GCLs was proposed. Preliminary results showed that the osmotic efficiency was 0.31 and 0.33, depending on the strain conditions [24]. Tests were also performed on exhumed samples for which the effective diffusivity and osmotic efficiency were measured to assess the effects of long-term exposure. The significance of chemical osmosis on a solute flux through bentonite barriers was studied in [27] using various parameters, including the osmotic efficiency, Peclet number, and osmotic number. The study indicates that chemical osmosis has only a minor influence on the solute transport.

In addition to the usual chemical osmosis (created by a gradient in the salt concentration, or in the chemical potential, across the barrier [5,9]), electro- and thermo-osmosis can also occur when the driving force is a gradient in the electric potential or temperature, respectively. For them, similar studies and results can be found [28,29,30,31,32]. Overviews of different approaches and models may be found in [33,34,35].

In this work, we would like to contribute to the study of osmosis, using an illitic clay as the investigated porous material and NaCl as the salt. We expect this material to have osmotic properties based on [10], where a mineral with a similar structure was studied. We describe a simple measurement apparatus proposed to measure the osmotic efficiency (or the reflection coefficient) *σ* and two other transport coefficients—the Darcy permeability and diffusion coefficient. We also estimate the uncertainties of these measurements. The apparatus and the model of the osmotic transport are similar to those introduced in [10] by Sherwood and Craster, although convenient modifications and simplifications are made in this study. We show that the osmotic transport plays a significant role for the studied illitic clay by measuring the transport coefficients and estimating the ratio of contributions coming from the individual transport mechanisms (diffusion, Darcy flow, and osmosis) to the total molar flow of water through the sample.

## 2. Experimental Procedure

**Figure 1:**
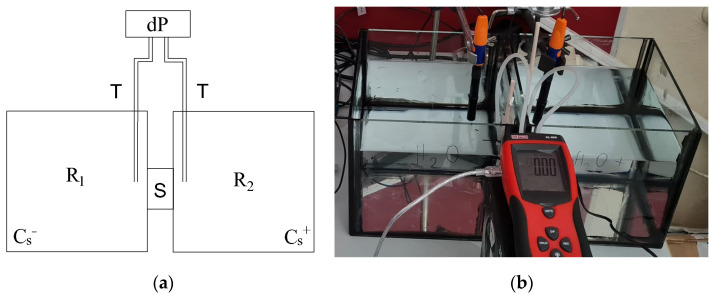
Apparatus for the measurement of the time evolution of the pressure difference across the sample: (**a**) a schematic representation, and (**b**) a photo.

The measurement apparatus was specifically designed to measure the pressure difference between the two solutions of salt concentrations C_s_^−^ and C_s_^+^ separated by a sample, S (see Figure 1). It consists of two reservoirs (R_1_ and R_2_) with dimensions 0.2 m × 0.2 m × 0.15 m, the volume of each reservoir thus being *V* = 6 × 10^−3^ m^3^. Both reservoirs have a circular hole with a diameter of 45 mm in one of their sides. The sample was sealed so as to be watertight to both circular holes from the external side of the reservoir, one to each side of the sample, using sanitary silicone to prevent any leaks. One reservoir (R_1_) was filled with deionized water and the other one (R_2_) with a solution of NaCl diluted in deionized water of a known concentration (10 wt.%). The pressure difference was measured by a differential manometer, dP (RS Pro RS-8890, RS Group plc, London, UK), through rubber tubes, T, with their ends located in both reservoirs at a distance of 50 mm from the center of the sample. The resolution of the manometer was ±5 Pa. The difference in the pressure was recorded regularly in 60 s intervals for the whole duration of the measurement, which takes about 120 h. The values which were used for our further analysis were obtained as averages of 60 measured points (30 before and 30 after the selected time).

The measured time evolution of the pressure difference was then fitted with Equation (4), given below, and the three transport coefficients, *k*, *D*, and *σ,* were calculated using the approach described in Section 3. Fitting of the experimental data was performed by non-linear regression using Levenberg–Marquardt algorithm in the OriginPro 2019b software.

**Figure 2:**
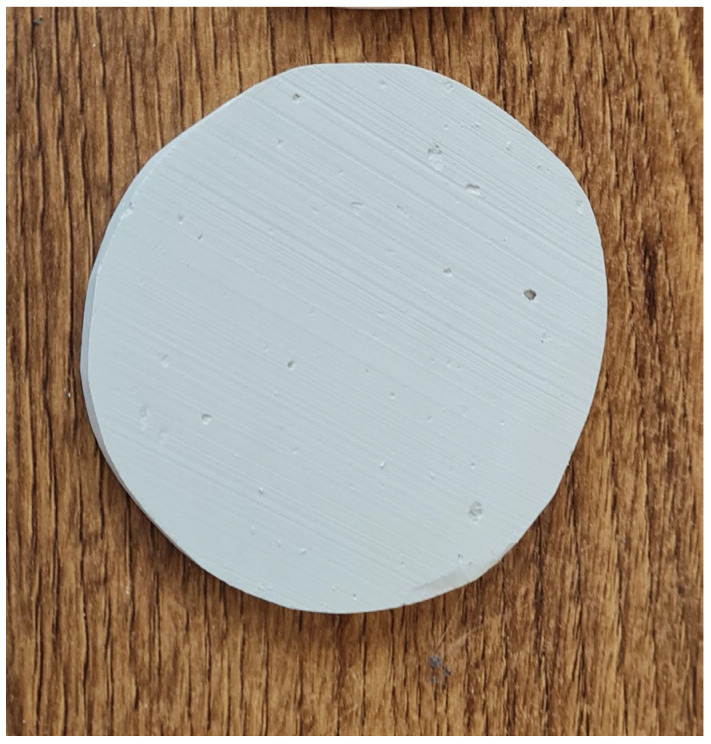
Photo of dry sample during preparation process. Surface irregularities, as well as shape and dimensions, are to be corrected using fine-gritted sandpaper.

Three samples were made from an illitic clay (from Füzerradvány, northeastern Hungary) which in its raw state contains mostly illite (~80 wt.%) and then quartz (~12 wt.%), montmorillonite (~4 wt.%), and orthoclase (~4 wt.%) [36]. The clay was ground using a planetary ball mill Retsch PM 100 (Retsch GmbH, Haan, Germany) and the ground substances were sieved to the point where their granulometry was smaller than 100 μm to ensure sufficient homogeneity of the clay. The sieved substance was mixed with deionized water in a 2:1 ratio to create a plastic mass that was formed into a cylindrical shape. The samples were then stored in an open container for free drying, which is slower than in a dryer, but causes fewer microcracks. After a two-week period, the dried samples were adjusted to the required thickness using medium-gritted sandpaper (see Figure 2) and then, to ensure that there are as few deviations from flatness as possible, their surfaces and edges were smoothed with sandpaper with a fine grit. Afterwards, the samples were fired to 900 °C with a heating rate of 5 K∙min^−1^ and then cooled freely in laboratory conditions. Each sample had a thickness of *h* = 5 mm and diameter of *d* = 45 mm, so that the area of the samples exposed to the solutions on both sides was *S* = 1.59 × 10^−3^ m^2^.

Sodium chloride (NaCl) was used as the salt. Its molecule dissociates into one cation of Na^+^ and one anion of Cl^−^, corresponding to *ν* = 2 ions in total. The initial difference in the salt mole fraction and mean salt mole fraction in equilibrium are Δ*x_s_^i^* = 2.99 × 10^−2^ and *x_s_*^0^ = 1.50 × 10^−2^, respectively. The molar masses of water and salt are *m_w_* = 0.018 kg∙mol^−1^ and *m_s_* = 0.0585 kg∙mol^−1^, respectively, and their molar volumes are *V_m_^w^* = 1.8 × 10^−5^ m^3^∙mol^−1^ and *V_m_^s^* = 1.66 × 10^−5^ m^3^∙mol^−1^, respectively. The surface area of the solutions in each reservoir was *A_b_* = 0.04 m^2^ and total number of water moles was *n_w_*^0^ = 332.96 mol. The measurements were performed in laboratory conditions at a temperature of *T* = 295 K.

The pore size distribution of the studied solid samples was measured by mercury porosimetry, using a Thermo Scientific (Waltham, MA, USA) Pascal 140 + 440 device. This device uses pressures from 0.01 Pa up to 400 kPa (with an inaccuracy of pressure detection less than 0.25%) for the impression of mercury into the pores of samples. The volume of mercury (the inaccuracy was less than 1% and the resolution was 0.0001 cm^3^) which was introduced into the pores of samples was measured for each applied pressure. Then, the size of pores was determined, keeping in mind that the applied pressure is inversely proportional to the size of pores according to the Washburn equation. This device can measure the pore size from 4 nm up to 100 um.

## 3. Theoretical Background

### 3.1. The Transport Model

The model which we use here is derived from the transport relations of water and ions dependent on the gradients in chemical and electric potentials of solutions [37] and Kedem–Katchalsky equations describing mass transport through membranes [38]. Since the electric potential is not applied here, the corresponding terms are absent. The changes in the chemical potential are expressed in terms of changes Δ*p* in the pressure and Δ*x*_s_ in the molar fraction of the salt. Assuming small variations from the equilibrium and taking into account the Onsager principle [39,40], the time evolution of Δ*p* and Δ*x*_s_ is described by the following pair of equations [10]:

(1) $$\frac{d\left(\Delta p\right)}{dt}=\alpha_{1}\Delta p+\alpha_{2}\Delta x_{s}\;,\;\frac{d\left(\Delta x_{s}\right)}{dt}=\beta_{1}\Delta p+\beta_{2}\Delta x_{s}\;.$$

These equations are identical to those derived by Sherwood and Craster in [10]. In fact, we followed their derivation with small changes reflecting differences between our and their measurement apparatuses. That is why the parameters *α*_1,2_ and *β*_1,2_ in Equation (1) are different from those in [10]. Namely, we have

(2) $$\alpha_{1}=-\frac{2Sg}{A_{b}}k\left[m_{s}\left(1-\sigma \right)x_{s}^{0}+m_{w}\left(1-x_{s}^{0}\right)\right]\;,\;\alpha_{2}=-\frac{2Sg}{A_{b}}\left[\left(1-\sigma \right)D\left(m_{s}-\frac{V_{m}^{s}}{V_{m}^{w}}m_{w}\right)-\sigma \nu RTk\frac{m_{w}}{V_{m}^{w}}\right]\;,\;\beta_{1}=-\frac{2Skx_{s}^{0}}{n_{w}^{0}}\left(x_{s}^{0}-\sigma \right)\;,\;\beta_{2}=-\frac{2S}{n_{w}^{0}}\left\{\left(1-\sigma \right)D+\frac{x_{s}^{0}}{V_{m}^{w}}\left[\left(1-\sigma \right)DV_{m}^{s}+\sigma \nu RTk\right]\right\},$$

where *g* = 9.81 m∙s^−2^ is the gravitational acceleration and *R* = 8.31 J∙mol^−1^∙K^−1^ is the universal gas constant. The coefficients *D* and *k* are proportional to the salt diffusivity *D_s_*, water diffusivity *D_w_*, and molar Darcy permeability *k_D_*, respectively, as

(3) $$D_{s}=V_{m}^{w}hD,\;\;\;\;\;\;D_{w}=V_{m}^{s}hD,\;\;\;\;\;\;k_{D}=\eta V_{m}^{w}hk.$$

In this study, we shall consider only the time evolution of the pressure difference Δ*p*, because (a) the measurement of the pressure difference is easier and more straightforward than the measurement of the molar fractions difference and (b) the solution of Equation (1) in terms of Δ*p* contains only three fitting parameters instead of four in the case of Δ*x*_s_. The time evolution of Δ*p* is given as the solution of Equation (1) and can be written as

(4) $$\Delta p(t)=A_{1}\left(e^{-A_{2}t}-e^{-A_{3}t}\right),$$

with

(5) $$A_{1}=\frac{\alpha_{2}\Delta x_{s}^{i}}{Y},\;\;\;\;\;\;A_{2}=\frac{\alpha_{1}+\beta_{2}+Y}{2},\;\;\;\;\;\;A_{3}=\frac{\alpha_{1}+\beta_{2}-Y}{2},$$

where

(6) $$Y=\sqrt{\alpha_{1}^{2}+4\alpha_{2}\beta_{1}-2\alpha_{1}\beta_{2}+\beta_{2}^{2}}$$

and Δ*x_s_^i^* is initial difference in mole fractions of solutions. As soon as the values of parameters *A*_1_, *A*_2_, and *A*_3_ are known (from fitting to experimental data), we can think of Equation (5) as a system of three equations that can be solved in terms of three studied transport parameters, *k*, *D*, and *σ*.

Finally, the molar flow of water from reservoir R_1_ to reservoir R_2_ is given as [10]

(7) $$f_{w}=f_{D}-f_{o}-f_{d},\;\;$$

where each term on the right side of the equation is defined as

(8) $$f_{D}=\left(1-x_{s}^{0}\right)k\Delta p,\;\;\;\;\;\;f_{o}=\frac{\sigma \nu RTk}{V_{m}^{w}}\Delta x_{s}^{i},\;\;\;\;\;\;f_{d}=\frac{\left(1-\sigma \right)DV_{m}^{s}}{V_{m}^{w}}\Delta x_{s}^{i}\;.$$

From Equation (8), we see that each of the three terms *f_D_*, *f_o_*, and *f_d_* corresponds to a different transport mechanism. Indeed, the first term *f_D_* is due to the Darcy flow (when Δ*x*_s_^i^ = 0, only this term remains in Equation (7), which then expresses Darcy’s law), the second term *f_o_* is due to osmosis (only this term remains in Equation (7) for Δ*p* = 0 Pa and *σ* = 1 when osmosis is the only possible mechanism of water flow), and the third term *f_d_* is due to diffusion (when Δ*p* = 0 Pa and *σ* = 0, only this term remains in Equation (7), which thus expresses Fick’s law of diffusion). The negative signs in front of the second and third terms in Equation (7) show that the Darcy flow has a direction that is opposite to the direction of osmosis and diffusion.

### 3.2. Evaluation of Uncertainties

Measurements are in general not completely accurate, but they always include some sort of uncertainty. According to the guide GUM [41], two basic types of uncertainties, *A* and *B*, are present during the measurement. Type *A* uncertainties are evaluated statistically from repeated measurements of the same parameter. The relevant statistical parameter, which describes the statistical dispersion of measured data, is the standard deviation *s*. Using this parameter, the statistical uncertainty (type *A* uncertainty), *u*_A_, of a parameter *x* is

(9) $$u_{A}\left(x\right)=\frac{s}{\sqrt{n}}=\sqrt{\frac{\sum_{i=1}^{n}{\left(x_{i}-\bar{x}\right)}^{2}}{n(n-1)}},$$

where $\bar{x}$ is the arithmetic average calculated from *n* measurements of the parameter *x* yielding the values *x_i_* (*i* = 1, …, *n*).

The other type of uncertainty, type *B*, is then estimated from the calibration certificates, information from manufacturers, or the apparatus resolution. From these, information about the maximum admissible error (MAE) of the measurement apparatus, or at least its resolution, *r*, can be extracted. The type *B* uncertainty is then calculated from either of them as follows [41]:

(10) $$u_{B}\left(x\right)=\frac{MAE}{\sqrt{3}},\;\;\;\;\;\;u_{B}\left(x\right)=\frac{r}{2\sqrt{3}}.$$

If there are multiple sources of the uncertainty, i.e., type *A* and type *B* uncertainty, or the studied quantity, *x*, is measured indirectly and calculated from *m* directly measured parameters, *y_i_* (*i* = 1, …, *m*), which are independent, and there is no correlation between them, they can be combined using the following equation [41]:

(11) $$u_{c}\left(x\right)=\sqrt{\sum_{i=1}^{m}{\left(\frac{\partial x}{\partial y_{i}}\right)}^{2}u_{c}{\left(y_{i}\right)}^{2}}.$$

## 4. Results

**Figure 3:**
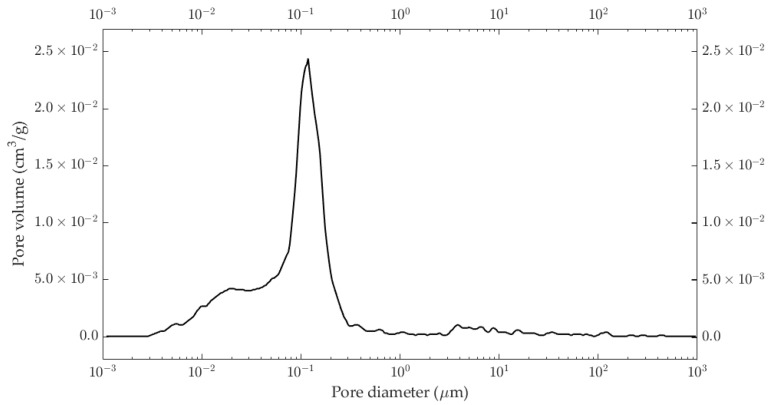
Pore size distribution of the illitic samples used in this work.

The apparent density of the studied samples was around 1.86 g∙cm^−3^ with an open porosity of around 35%. The pore size distributions of the studied illitic samples are plotted in Figure 3. It shows that most pores had a diameter of around 0.1 μm.

### 4.1. Time Evolutions

**Figure 4:**
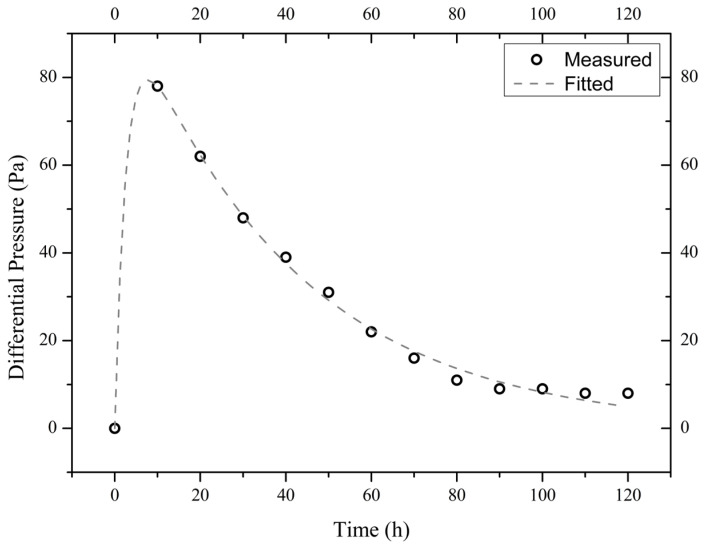
Measured and fitted time evolutions of the differential pressure across the sample I900-1 exposed to a 10 wt.% NaCl solution.

**Figure 5:**
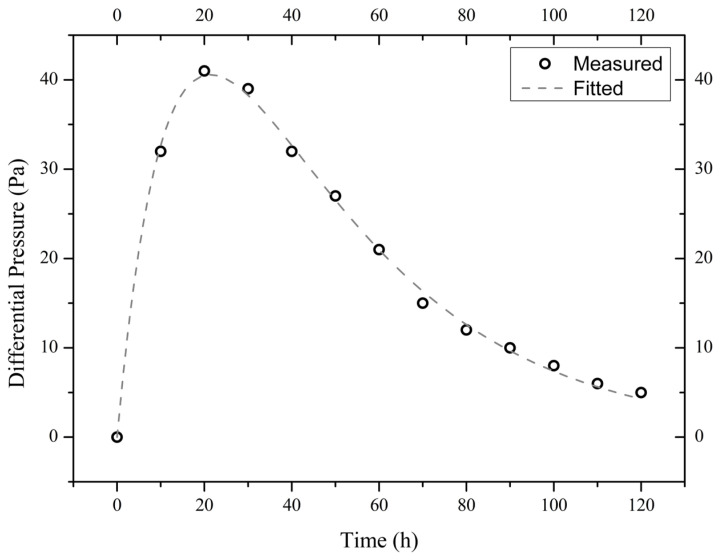
Measured and fitted time evolutions of the differential pressure across the sample I900-2 exposed to a 10 wt.% NaCl solution.

**Figure 6:**
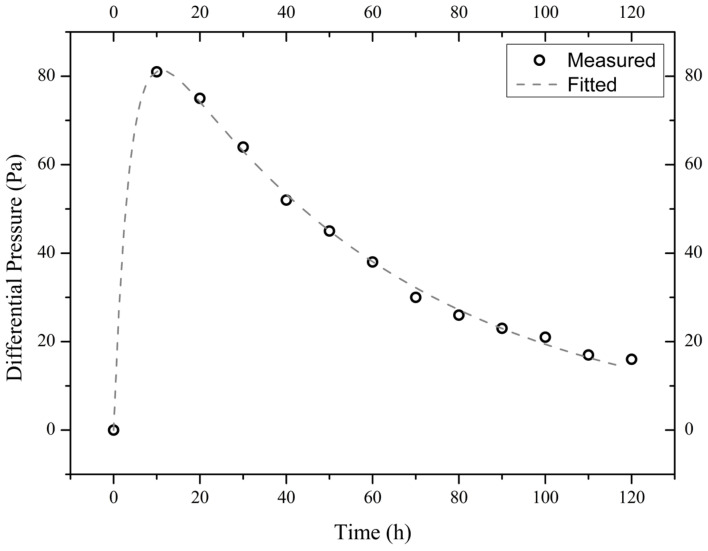
Measured and fitted time evolutions of the differential pressure across the sample I900-3 exposed to a 10 wt.% NaCl solution.

The measured time evolutions of the pressure difference of the three illite samples are shown in Figure 4, Figure 5 and Figure 6. These experimental data are best fitted (by non-linear regression analysis) with the theoretical formula from Equation (4), thus obtaining the values of parameters *A*_1_, *A*_2_, and *A*_3_ (see Table 1).

Using these values of *A_i_*, we then solved the set of three equalities in Equation (5) to get the three transport coefficients *σ*, *k*, and *D*. Finally, Equation (3) was used to calculate (a) the effective salt and water diffusivities *D_s_* and *D_w_*, respectively, and (b) the Darcy permeability *k_D_*, assuming that the viscosity of water is approximately *η* = 10^−3^ Pa s. The values of all of these parameters are given in Table 1.

Table 1 shows significant differences in the values of the transport parameters *D_s_*, *D_w_*, and *k_D_* calculated from measurements of different samples. These differences could be attributed to slightly different pore structures, such as tortuosity. These differences should be minimized if the samples are prepared more consistently, e.g., by using suitable pressure.

Since the radius of particles is usually smaller than 1 nm, we see that the pore size is not the go-to parameter to explain the osmotic behavior of the studied material. Also, the values of the osmotic coefficient *σ* is in the order of 10^−4^ for every sample, meaning that only about 0.01% of the salt particles are restricted from the transport through the membrane. Hence, another explanation is required. This effect likely occurs due to the creation of low local electrostatic fields that repel electrically charged salt ions. It may also be caused by the other properties related to porosity, i.e., tortuosity. Measured effective diffusivities are in the order of 10^−7^ m^2^∙s^−1^, which corresponds with other porous materials.

### 4.2. Flow of Water

The molar flow of water *f_w_* through the studied samples can be calculated from Equations (7) and (8) using the values of coefficients listed in Table 1. In addition to its total value, however, we also calculate the values of its three individual components, *f_D_*, *f_o_*, and *f_d_,* which correspond to the Darcy flow, osmosis, and diffusion, using Equation (8). To calculate the Darcy flow *f_D_*, the pressure difference Δ*p* is needed. Since Δ*p* is time dependent, we take its maximum measured for each sample to get a maximal value of the Darcy flow *f_D_*. The resulting values of the three flows are given in Table 2.

Table 2 shows that osmosis is the dominant contribution to the total molar flow of water through the measured samples. Even though the total value of the molar flow varies greatly between the samples, the ratio between its components remains almost constant: the contribution of the Darcy flow *f_D_* is 1–5%, diffusion makes 11–13% of the total flow, and 82–88% of the flow is due to osmosis. Even after considering the deviation of up to ~22%, as suggested in Table 1 for the coefficients *σ*, *k,* and *D*, osmosis is still a dominant contribution to the total flow.

The deviations in the total flow are related to slight micro-structural differences in the samples. Nevertheless, the ratio of the specific transport modes of the water through the sample remains similar in all three cases. The most significant result is that the contribution coming from the osmotic mode of transport is convincingly the highest in all three samples.

### 4.3. Uncertainties

All quantities used in the calculations appear in the expressions for *α*_1,2_ and *β*_1,2_ from Equation (2). Some of the quantities (such as the molar volumes and molar masses) are tabulated, and their uncertainties are therefore neglected. The uncertainties of the remaining quantities, such as the exposed area of the sample; surface area of the reservoirs; initial and equilibrium molar fractions of the salt; number of water moles; temperature during the measurements; and the uncertainties in the fitting parameters *A*_1_, *A*_2_, and *A*_3_, are evaluated below.

The exposed area of the sample is measured indirectly, where the directly measured quantities are the diameter *d* of the holes made in the sides of the two reservoirs. The diameter was measured five times for each reservoir, given the total of *n* = 10 values. Using Equation (9), the type *A* uncertainty of the diameter is *u_A_*(*d*) = 4.4 × 10^−5^ m. The diameter was measured using a digital caliper with a resolution of *r* = 0.1 mm. The type *B* uncertainty calculated from Equation (10) is therefore *u_B_*(*d*) = 2.0 × 10^−5^ m. The combined standard uncertainty in the measurement of the diameter is *u*_c_(*d*) = 4.9 × 10^−5^ m, according to Equation (11). The exposed area of the sample is then calculated from the diameter as *S* = *πd*^2^/4. Using Equation (11) now, the total uncertainty in the measurement of the exposed area of the sample is *u*_c_(*S*) = 3.5 × 10^−6^ m^2^. Similarly, the surface area *A_b_* = *a∙b* of the reservoir was measured indirectly by measuring its length, *a*, and width, *b.* Both dimensions were measured 10 times, with *u_A_*(*a*) *=* 5.0 × 10^−4^ m and *u_A_*(*b*) = 5.4 × 10^−4^ m, by the same caliper as the diameter of the holes in the reservoir, so that the type *B* uncertainty is the same: *u_B_*(*a*) = *u_B_*(*b*) = *u_B_*(*d*). Hence, the combined uncertainties are *u*_c_(*a*) = 5.0 × 10^−4^ m and *u*_c_(*b*) = 5.4 × 10^−4^ m, yielding the combined uncertainty in the surface area *u_c_*(*A_b_*) = 1.5 × 10^−4^ m^2^ from Equation (11).

Since one of the reservoirs is filled with pure deionized water, the initial difference in the mole fraction of salt in the solution and its equilibrium value are related to each other as Δ*x_s_^i^* = 2*x_s_*^0^. The uncertainties in their measurement are therefore presented together. The mole fraction of the solution may be expressed as

(12) $$x_{s}=\frac{\frac{M_{s}}{m_{s}}}{\frac{M_{w}}{m_{w}}+\frac{M_{s}}{m_{s}}}=\frac{\frac{M_{s}}{m_{s}}}{\frac{M_{s}}{m_{s}}+\frac{V\rho }{m_{w}}},$$

where *M_s_* is total mass of the salt diluted in the water (which is 600 g) and *ρ* = 1 g cm^−3^ is the density of deionized water. The volume of water is measured only once by the apparatus with a resolution of *r_V_* = 5 × 10^−5^ m^3^, so that the uncertainty in the volume measurement, according to Equation (10), is *u*_c_(*V*) = *u*_B_(*V*) = 1.0 × 10^−5^ m^3^. Similarly, the total mass of diluted salt is measured only once on scales with an MAE = 10^−3^ kg, and so its uncertainty is *u*_c_(*M*_s_) = *u*_B_(*M*_s_) = 5.8 × 10^−4^ kg. The combined uncertainty in the measurement of the initial difference in the mole fraction of salt is then *u_c_*(Δ*x_s_^i^*) = 5.6 × 10^−5^, and in equilibrium, *u_c_*(*x_s_*^0^) = 3.8 × 10^−5^.

The total number of water moles is calculated as the ratio of the total volume to the molar volume of the water, *n_w_*^0^ = *V*/*V_m_^w^*. Since *u*_c_(*V*) is already evaluated, Equation (11) gives the uncertainty in the total number of water moles as *u_c_*(*n_w_*^0^) = 0.57. The temperature varied by ±2 K during the measurements, so the *u_c_*(*T*) = 2 K.

Finally, the parameters obtained by fitting the measured data are considered. The software used to fit the data provided some statistically derived deviations for each parameter. For the purpose of this paper, these would be categorized as type *A* uncertainties. Type *B* uncertainties are problematic, because the directly measured parameter, pressure difference, is not included anywhere in the calculations. To estimate the influence of the apparatus on the parameters *A*_1_, *A*_2_, and *A*_3_, the measured data were varied within the range of the apparatus resolution to assess its effect on *A_i_*. It was estimated that as the data were varied, *A_i_* changed within the interval of ±2*u_A_* of its respective value. These parameters and their uncertainties are different for each measurement. Uncertainties of these parameters for the I900-1 sample are *u_c_*(*A*_1_) = 4.09 Pa, *u_c_*(*A*_2_) = 2.9 × 10^−7^ s^−1^, and *u_c_*(*A*_3_) = 8.2 × 10^−6^ s^−1^. For the I900-2 sample, the obtained uncertainties have values of *u_c_*(*A*_1_) = 21.81 Pa, *u_c_*(*A*_2_) = 6.5 × 10^−7^ s^−1^, and *u_c_*(*A*_3_) = 2.4 × 10^−6^ s^−1^. Lastly, fitting of the measurement of sample I900-3 gives the uncertainties *u_c_*(*A*_1_) = 3.90 Pa, *u_c_*(*A*_2_) = 2.04 × 10^−7^ s^−1^, and *u_c_*(*A*_3_) = 1.33 × 10^−6^ s^−1^. The combined uncertainties of the measured parameters are summarized in Table 1.

Due to the nature of this experiment, repeated measurements of identical samples are inappropriate, since salt particles may settle in the pores of the samples and, therefore, alter the progress of consecutive measurements. Hence, effects of the repeated measurements are not included in the evaluation of uncertainties of the measurement.

## 5. Conclusions

An apparatus for the measurement of osmosis in porous materials and a model for the determination of osmotic contribution to the total flow of water were described in this study. The apparatus is based on the measurement of the differential pressure across the bulk porous sample exposed to the solution with different concentrations on each side. The model predicts an exponential type of the time dependence of the differences of pressure and salt concentration across a sample. Both the apparatus and model were motivated by and are simplified modifications of those proposed by Sherwood and Craster [10].

We performed measurements for three samples made from an illitic clay fired to 900 °C. Although the transport parameters, as well as the total volume of molar flow, show differences between the studied samples, the ratio of the individual components of the total flow remains very similar in each sample. Our results show that diffusion and Darcy flow contribute to the total flow of water by about 12% and 3%, respectively, while osmosis is responsible for about 86% of the total flow. Thus, osmotic flow is the dominant type of water flow for the studied samples, in contrast to the flow of solute across the Na–bentonite-based GCLs shown in [27]. This main conclusion remains true even when we consider the rather large uncertainties of the measured values of parameters of up to 22%. Therefore, the presented technique can be applied to determine the role of osmosis in the flow of water in porous materials and to estimate to what extent a material behaves as a semipermeable membrane.

In the future, we plan to extend the studies of osmosis to a wider range of materials and to improve the resolution of our apparatus. We also plan to use different methods of sample preparation to get more uniform samples, which should result in smaller differences between individual transport parameters.

## Figures and Tables

**Table 1:** Summary of values and uncertainties in measurement of transport parameter *σ*, *k*, and *D*.

Measured Quantity		Value	*u*_c_	Unit	Relative *u*_c_ (%)
Exposed area *S*		1.59 × 10^−3^	3.5 × 10^−6^	m^2^	0.2
Surface area of the reservoir *A_b_*		4 × 10^−2^	1.5 × 10^−4^	m^2^	0.4
Sample thickness		5 × 10^−3^	1 × 10^−4^	m	2.0
Total number of water moles *n_w_*^0^		332.96	0.57	–	0.2
Temperature *T*		295	2	K	0.7
Initial mole fraction difference Δ*x_s_^i^*		2.99 × 10^−2^	5.6 × 10^−5^	–	0.2
Mole fraction in equilibrium *x_s_*^0^		1.50 × 10^−2^	3.8 × 10^−5^	–	0.3
I900-1	*A*_1_	106.83	4.09	Pa	3.8
	*A*_2_	7.31 × 10^−6^	2.9 × 10^−7^	s^−1^	4.0
	*A*_3_	8.07 × 10^−5^	8.2 × 10^−6^	s^−1^	10.2
	Osmotic efficiency *σ*	3.99 × 10^−4^	4.7× 10^−5^	–	11.8
	Molar membrane permeability *k*	5.03 × 10^−4^	2.0 × 10^−5^	mol∙Pa^−1^∙s^−1^∙m^−2^	4.0
	Molar salt diffusivity *D*	7.53	0.76	mol∙m^−2^∙s^−1^	10.1
	Effective salt diffusivity *D_s_*	6.73 × 10^−7^	7.0 × 10^−8^	m^2^∙s^−1^	10.4
	Effective water diffusivity *D_w_*	6.21 × 10^−7^	6.4 × 10^−8^	m^2^∙s^−1^	10.3
	Darcy permeability *k_D_*	0.046	0.002	Darcy	4.3
I900-2	*A*_1_	116.49	21.81	Pa	18.3
	*A*_2_	7.63 × 10^−6^	6.5 × 10^−7^	s^−1^	8.5
	*A*_3_	2.04 × 10^−5^	2.4 × 10^−6^	s^−1^	11.8
	Osmotic efficiency *σ*	8.1 × 10^−5^	1.8 × 10^−5^	–	22.2
	Molar membrane permeability *k*	5.25 × 10^−4^	4.5 × 10^−5^	mol∙Pa^−1^∙s^−1^∙m^−2^	8.6
	Molar salt diffusivity *D*	1.94	0.22	mol∙m^−2^∙s^−1^	11.3
	Effective salt diffusivity *D_s_*	1.74 × 10^−7^	2.0 × 10^−8^	m^2^∙s^−1^	11.5
	Effective water diffusivity *D_w_*	1.61 × 10^−7^	1.9 × 10^−8^	m^2^∙s^−1^	11.8
	Darcy permeability *k_D_*	0.048	0.004	Darcy	8.3
I900-3	*A*_1_	104.62	3.90	Pa	3.7
	*A*_2_	4.86 × 10^−6^	2.0 × 10^−7^	s^−1^	4.1
	*A*_3_	7.41 × 10^−5^	1.3 × 10^−5^	s^−1^	17.5
	Osmotic efficiency *σ*	5.54 × 10^−4^	1.05 × 10^−4^	–	19.0
	Molar membrane permeability *k*	3.35 × 10^−4^	1.4 × 10^−5^	mol∙Pa^−1^∙s^−1^∙m^−2^	4.2
	Molar salt diffusivity *D*	6.91	1.21	mol∙m^−2^∙s^−1^	17.5
	Effective salt diffusivity *D_s_*	6.22 × 10^−7^	1.1 × 10^−7^	m^2^∙s^−1^	17.7
	Effective water diffusivity *D_w_*	5.74 × 10^−7^	1.0 × 10^−7^	m^2^∙s^−1^	17.4
	Darcy permeability *k_D_*	0.031	0.001	Darcy	3.2

**Table 2:** Components of the molar flow of water through the samples.

Sample	Darcy Flow	Osmosis	Diffusion	Ratio
	(×10^−3^ mol∙m^−2^∙s^−1^)			(%:%:%)
I900-1	38.7	1634.5	207.6	2.1:86.9:11.0
I900-2	21.2	346.3	53.5	5.0:82.3:12.7
I900-3	26.7	1511.5	190.4	1.6:87.4:11.0

## Data Availability

The original contributions presented in this study are included in the article. Further inquiries can be directed to the corresponding author.

## References

[1] Borg F.G. (2003). What Is Osmosis? Explanation and Understanding of a Physical Phenomenon. arXiv.

[2] Grathwohl P. (1998). Diffusion in Natural Porous Media: Contaminant Transport, Sorption/Desorption and Dissolution Kinetics. Topics in Environmental Fluid Mechanics.

[3] Mitchell J.K., Soga K. (2005). Fundamentals of Soil Behavior.

[4] Malusis M.A., Shackelford C.D. (2002). Chemico-Osmotic Efficiency of a Geosynthetic Clay Liner. J. Geotech. Geoenviron. Eng..

[5] Olsen H.W. (1972). Liquid Movement Through Kaolinite under Hydraulic, Electric, and Osmotic Gradients. Am. Assoc. Pet. Geol. Bull..

[6] Neuzil C.E. (2000). Osmotic Generation of ‘Anomalous’ Fluid Pressures in Geological Environments. Nature.

[7] Murray H.H. (2007). Structure and composition of the clay minerals and their physical and chemical properties. Dev. Clay Sci..

[8] Grim R.E. (1968). Clay Mineralogy.

[9] Fritz S.J. (1986). Ideality of Clay Membranes in Osmotic Processes: A Review. Clays Clay Miner..

[10] Sherwood J.D., Craster B. (2000). Transport of Water and Ions Through a Clay Membrane. J. Colloid Interface Sci..

[11] Bader S., Kooi H. (2005). Modelling of Solute and Water Transport in Semi-Permeable Clay Membranes: Comparison with Experiments. Adv. Water Resour..

[12] Garavito A.M., Kooi H., Neuzil C.E. (2006). Numerical Modeling of a Long-Term in Situ Chemical Osmosis Experiment in the Pierre Shale, South Dakota. Adv. Water Resour..

[13] Garavito A.M., De Cannière P., Kooi H. (2007). In Situ Chemical Osmosis Experiment in the Boom Clay at the Mol Underground Research Laboratory. Phys. Chem. Earth.

[14] Mokni N., Olivella S., Valcke E., Mariën A., Smets S., Li X. (2011). Deformation and Flow Driven by Osmotic Processes in Porous Materials: Application to Bituminised Waste Materials. Transp. Porous Media.

[15] Kang J.B., Shackelford C.D. (2010). Membrane Behavior of Compacted Clay Liners. J. Geotech. Geoenviron. Eng..

[16] Mokni N., Olivella S., Alonso E.E.E. (2010). Swelling in Clayey Soils Induced by the Presence of Salt Crystals. Appl. Clay Sci..

[17] Mokni N., Olivella S., Li X., Smets S., Valcke E. (2008). Deformation Induced by Dissolution of Salts in Porous Media. Phys. Chem. Earth.

[18] Guarena N., Dominijanni A., Manassero M. (2024). Reflection coefficient of a natural sodium bentonite in aqueous mixed electrolyte solutions: Positive and negative anomalous osmosis. Can. Geotech. J..

[19] Zheng J.Z., Zhang Z.H., Tian G.L. (2022). Membrane Behavior of Kaolin Bentonite Mixtures. J. Geotech. Geoenviron. Eng..

[20] Song Z.Y., Wei C.F., Cai G.Q., Zhang Z.H., Du X.L. (2023). Membrane behavior of clay considering the effect of fixed charges. Sci. Total Environ..

[21] Fritz C.J., Scalia J., Shackelford C.D., Malusis M.A. (2020). Determining Maximum Chemico-Osmotic Pressure Difference across Clay Membranes. J. Geotech. Geoenviron. Eng..

[22] Zhang Z.H., Yang H.W., Song Z.Y. (2024). Membrane behavior of clay under mixed solution conditions. Sci. Total Environ..

[23] Zhang Z.H., Yang H.W., Song Z.Y. (2023). Membrane efficiency model of clay under multiionic conditions. J. Clean. Prod..

[24] Mazzieri F., Bernardo D. Chemico-Osmotic Coefficients of Geosynthetic Clay Liners under Different Confinement Conditions. Proceedings of the Geo-Congress on Sustainable Infrastructure Solutions from the Ground Up.

[25] Bernardo D., Domizi J., Fratalocchi E., Mazzieri F. Chemico-Osmotic efficiency of geosynthetic clay liners: Testing apparatus and preliminary results. Proceedings of the 12th International Conference on Geosynthetics (12ICG).

[26] Tong S., Sample-Lord K.M., Rahman S.A.B., Yesiller N., Hanson J.L. Diffusion and membrane behavior of an exhumed geosynthetic clay liner. Proceedings of the 12th International Conference on Geosynthetics (12ICG).

[27] Malusis M.A., Scalia J., Norris A.S., Shackelford C.D. (2020). Effect of chemico-osmosis on solute transport in clay barriers. Environ. Geotech..

[28] Rosanne R., Paszkuta M., Tevissen E., Adler P.M. (2003). Thermodiffusion in Compact Clays. J. Colloid Interface Sci..

[29] Rosanne M., Paszkuta M., Adler P.M. (2006). Thermodiffusional Transport of Electrolytes in Compact Clays. J. Colloid Interface Sci..

[30] Soler J.M. (2001). The Effect of Coupled Transport Phenomena in the Opalinus Clay and Implications for Radionuclide Transport. J. Contam. Hydrol..

[31] Gonçalvès J., Trémosa J. (2010). Estimating Thermo-Osmotic Coefficients in Clay-Rocks: I. Theoretical Insights. J. Colloid Interface Sci..

[32] Trémosa J., Gonçalvès J., Matray J.M., Violette S. (2010). Estimating Thermo-Osmotic Coefficients in Clay-Rocks: II. In Situ Experimental Approach. J. Colloid Interface Sci..

[33] Zubair M.M., Saleem H., Zaidi S.J. (2023). Recent progress in reverse osmosis modeling: An overview. Desalination.

[34] Qasim M., Badrelzaman M., Darwish N.N., Darwish N.A., Hilal N. (2019). Reverse osmosis desalination: A state-of-the-art review. Desalination.

[35] Medved I., Černý R. (2013). Osmosis in Porous Media: A Review of Recent Studies. Microporous Mesoporous Mater..

[36] Húlan T., Trník A., Štubňa I., Bačík P., Kaljuvee T., Vozár L. (2015). Thermomechanical Analysis of Illite from Füzérradvány. Mater. Sci..

[37] Staverman A.J., Smith J.A.M. (1975). Physical Chemistry: Enriching Topics from Colloid and Surface Sciences.

[38] Kedem O., Katchalsky A. (1958). Thermodynamic Analysis of the Permeability of Biological Membranes to Non-Electrolytes. Biochim. Biophys. Acta.

[39] Onsager L. (1931). Reciprocal Relations in Irreversible Processes. I. Phys. Rev..

[40] Onsager L. (1931). Reciprocal Relations in Irreversible Processes. II. Phys. Rev..

[41] (2008). Evaluation of Measurement Data—Guide to the Expression of Uncertainty in Measurement.

